# L-cysteine mitigates busulfan-induced testicular injury through modulation of CBS/H_2_S axis

**DOI:** 10.3389/fcell.2025.1679330

**Published:** 2025-10-23

**Authors:** Song Liu, Bin Wei, Han Wei, Anran Xu, Lianbing Sheng, Xiangyang Sun, Yunling Dong, Huijun Yang

**Affiliations:** ^1^ Shandong Provincial Maternal and Child Health Care Hospital Affiliated to Qingdao University, Jinan, China; ^2^ Department of Reproductive Medicine, Key Laboratory of Birth Regulation and Control Technology of National Health Commission of China, Shandong Provincial Maternal and Child Health Care Hospital Affiliated to Qingdao University, Jinan, China; ^3^ Dalian Women and Children’s Medical Group, Reproductive and Genetic Medicine Center, Dalian, China; ^4^ Key Laboratory of Birth Regulation and Control Technology of National Health Commission of China, Shandong Provincial Maternal and Child Health Care Hospital Affiliated to Qingdao University, Jinan, China

**Keywords:** L-cysteine, CBS/H2S, oxidative stress, blood-testis barrier, PI3K/Akt/mTOR pathway

## Abstract

Global male infertility, characterized by decreased spermatogenesis and sperm quality, is a significant concern. L-cysteine, essential for hydrogen sulfide (H_2_S) synthesis, offers numerous biological advantages. However, the protective mechanisms of L-cysteine in treating spermatogenic dysfunction need further exploration. This study aims to examine L-cysteine’s protective effects on busulfan-induced testicular toxicity. Results show that administering L-cysteine at different doses (2.5, 5.0, and 10 mg/kg) led to notable improvements in final body weight, testis weight, sperm count, sperm motility, testosterone levels, and seminiferous tubule architecture. At a 5.0 mg/kg dosage, L-cysteine mitigated testicular injury by activating the CBS/H_2_S axis. Moreover, L-cysteine effectively reduced apoptosis and oxidative stress through Nrf2/HO-1 pathway activation. Various analyses demonstrated that L-cysteine enhanced the repair of the blood-testis barrier (BTB) disrupted by busulfan. Mechanistically, L-cysteine activated the PI3K/Akt/mTOR pathway in the testes. Notably, the CBS inhibitor AOAA reversed L-cysteine’s protective effects on busulfan-induced testicular damage. In summary, the study suggests that L-cysteine can safeguard against busulfan-induced spermatogenic dysfunction, apoptosis, oxidative stress, and BTB disruption by modulating the PI3K/Akt/mTOR pathway, hinging on CBS/H_2_S axis activation. These findings propose L-cysteine as a potential treatment for male infertility, particularly in individuals undergoing busulfan chemotherapy.

## Graphical Abstract

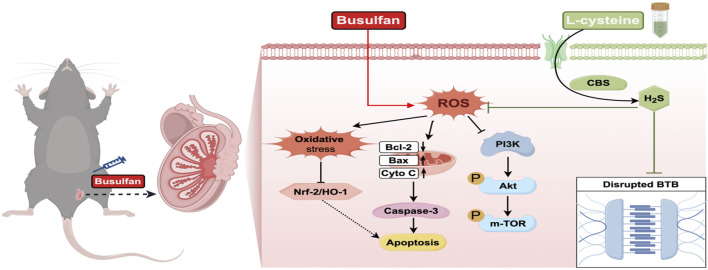

## 1 Introduction

Infertility is a prevalent global health concern, with approximately 15% of couples experiencing fertility issues, half of which are attributed to male factors. Male infertility primarily stems from impairments in spermatogenesis (Jafari et al., 2021). Spermatogenesis can be adversely affected by a range of factors, encompassing environmental elements, chemotherapeutic agents, oxidative stress, intense heat exposure and radiation (Tseng, 2022). Busulfan, a commonly used drug for treating chronic myelogenous leukemia in children and adults, has been found to negatively impact testicular tissue and interfere with sperm production in males, ultimately causing lower sperm counts and quality, which could potentially reduce male fertility (Huang et al., 2021). Increased oxidative stress toxicity and apoptosis are linked to the impairment of male reproductive function caused by busulfan (Abarikwu et al., 2022a; Ibrahim et al., 2021). Moreover, several researches have shown that busulfan interferes with the blood-testis barrier (BTB) by causing disarray and changing the way important BTB junction elements like ZO-1, occludin, claudin-11, connexin 43, β-catenin, and N-cadherin are expressed (Liu et al., 2021; Jiang et al., 2022; Zhao et al., 2022). Consequently, the urgent need to devise a medical preventive strategy to safeguard testicular function against chemotherapy-induced harm in patients undergoing busulfan treatment has emerged as a significant global apprehension.

Hydrogen sulfide (H_2_S) is now recognized as an important gas signaling molecule, along with nitric oxide and carbon monoxide, and is understood to be essential in regulating different cellular processes under physiological and pathological conditions (Silva-Velasco et al., 2024; Oliv et al., 2023; Xuan et al., 2022). This antioxidant property of H_2_S is particularly beneficial in reducing pathological oxidative stress associated with testicular torsion injury and enhancing spermatogenic activity in the lumen of most seminiferous tubules (Yuksel et al., 2022). Also, the administration of exogenous H_2_S has been shown to safeguard testicular function by suppressing the inflammatory and oxidative consequences in mice treated with lipopolysaccharide (Wang et al., 2018). Importantly, H_2_S has the ability to attenuate reproductive toxicity induced by Nanoplastics by mitigating mitochondrial apoptosis and excessive autophagy (Li et al., 2022). Nevertheless, the potential therapeutic value of H_2_S for testicular dysfunction caused by busulfan has not been elucidated.

Compounds that release H_2_S in response to a specific trigger, known as H_2_S donors, have been used in studies to show the therapeutic advantages of administering H_2_S in different experimental settings (Hao et al., 2021; Lee and Deng, 2015). L-cysteine serves as a substrate for the creation of H_2_S, which naturally produced by cystathionine-β-synthase (CBS), cystathionine-γ-lyase (CSE) and 3-mercaptopyruvate sulfurtransferase (3-MST) through desulfurization (Bełtowski, 2015). L-cysteine was designed to model the endogenous, physiological H_2_S production (Liu et al., 2017). For instance, the administration of L-Cysteine has been shown to reduce harm to myenteric neurons and enteric neural precursor cells caused by intestinal ischemia/reperfusion (Gao et al., 2022). A prior investigation from our research group demonstrated that L-Cysteine can provide neuroprotection against neonatal hypoxic-ischemic injury in neonates, partly attributed to its anti-apoptotic properties and reduction of oxidative stress via the CBS/H_2_S system (Liu et al., 2017). However, to our knowledge, it is still not clear whether or not L-cysteine can attenuate apoptosis and oxidative stress in busulfan-exposed testicular toxicity, or which H_2_S-producing enzymes are activated by L-cysteine.

In order to validate our theory, this study concentrated on interrogating the therapeutic potential and underlying mechanism of L-cysteine on spermatogenesis dysfunction, apoptosis, oxidative damage, and BTB disruption in busulfan-triggered testicular damage in mice by modulating the PI3K/Akt/mTOR pathway. More specifically, we investigated how L-cysteine can protect by increasing the activity of the CBS/H_2_S axis.

## 2 Materials and methods

### 2.1 Animals and experimental design

Male C57BL/6J mice, 8 weeks old and weighing 16–20 g, were obtained from Jinan pengyue Laboratory Animal Breeding Co., Ltd. All mice are fed in the Specific Pathogen Free (SPF) mouse house. All mice were randomly assigned into five groups, namely: (1) NC group (*n =* 10): mice were intraperitoneally administered an equal volume of vehicle (saline) four times over the course of 1 week; (2) NC+L-C10 group (*n =* 10): mice were intraperitoneally administered an equal volume of L-Cysteine (10.0 mg/kg; dissolved in distilled water; Cat# 168149, Sigma, United States) four times over the course of 1 week; (3) BSF group (*n =* 10): mice were received a single dose of 30 mg/kg busulfan (dissolved in dimethyl sulfoxide and added with 100 µL of distilled water; Cat# B2635, Sigma, United States); (4) BSF+L-C group (*n =* 30): Following the administration of busulfan, the mice were randomly allocated into three groups (*n =* 10) and subsequently intraperitoneally injected with varying doses of L-Cysteine (2.5/5.0/10 mg/kg) on four times over the course of 1 week; (5) BSF+L-C+AOAA group (*n =* 10): aminooxyacetic acid (AOAA; 10.0 mg/kg; dissolved in distilled water; Cat# C13408, Sigma, United States) was administered via intraperitoneal injection 1 h prior to the administration of L-Cysteine, followed by four additional doses of L-Cysteine (5.0 mg/kg) over the span of 1 week. Afterward, the left testis of every mouse was frozen at −80 °C, the right testis was fixed in 4% paraformaldehyde (PFA) and blood from mice were centrifuged to obtain serum. Sperm count and motility were evaluated following established methodologies, utilizing a sample size of six mice per group. The sperm concentration was quantified and reported as 10^6^/epididymis.

### 2.2 Measurement of testis H_2_S level

The quantification of H_2_S production in testis tissues was conducted in accordance with established procedures (Qabazard et al., 2021). In short, testis tissues were homogenized in a cold potassium phosphate buffer with the composition of 100 mM potassium phosphate, 10 mM L-cysteine, and 2 mM pyridoxal 5′-phosphate. Additionally, 1% zinc acetate was included as the trapping solution. The reaction was halted by the addition of 10% trichloroacetic acid, followed by a reaction with N, N-dimethyl-p-phenylenediamine sulfate and FeCl_3_ in 1.2 M HCl. Subsequent to a 20-min incubation, the absorbance of the final solution at 670 nm was measured using a spectrophotometer.

### 2.3 Analysis of testosterone levels in mouse serum

Serum testosterone (T) levels were measured by an enzyme-linked immunosorbent assay (ELISA) kit (Cat# E-OSEL-M0003, Elabscience Biotechnology, Wuhan, China) following the instructions provided in the user manual.

### 2.4 Oxidative stress marker analysis

Assay kits from Nanjing Jiancheng Biological Engineering Institute in China were used to measure oxidative stress markers in testis tissues, such as Superoxide Dismutase (SOD; Cat# A001-3-2), Glutathione Peroxidase (GSH-Px; Cat# A005-1-2), Catalase (CAT; Cat# A007-2-1) activity as well as Malondialdehyde (MDA; Cat# A003-1-2) production.

Dihydroethidium (DHE) staining was used to evaluate the generation of reactive oxygen species (ROS) in testicular tissues. Fresh, unfixed, frozen testis sections (10 μm thick) were incubated with 10 μM DHE from Beyotime in Shanghai, China, at 37 °C for 30 min right after being prepared. Afterward, the slides were rinsed with cold PBS and examined using a fluorescence microscope (Olympus BX53, Tokyo, Japan). The intensity of fluorescence was analyzed quantitatively with the ImageJ software created by the National Institute of Mental Health in Bethesda, Maryland, United States.

### 2.5 Transmission electron microscopy (TEM)

Fragments of testicular tissue, each measuring 1 mm^3^, were extracted from mice 24 h post-reperfusion and then preserved in 2.5% glutaraldehyde at 4 °C for the duration of the night. The samples of testicular tissue were subjected to a sequence of procedures that involved cleansing, stabilizing, removing moisture, encasing, and treating with a buffer solution, prior to being sliced into extremely thin sections. The sections were then observed and photographed using a Hitachi H-7500 TEM.

### 2.6 Histopathological assays

Assessment of testicular histology was conducted as previously described (Hong et al., 2021). The testicular tissues, which were isolated and cut into 5 µm-thick sections, were subjected to a sequence of steps that involved slicing, removing paraffin, drying out, and staining with hematoxylin and eosin (H&E) in order to assess abnormal changes using light microscopy (Olympus BX53, Tokyo, Japan).

### 2.7 TUNEL staining

The presence of apoptotic cells in the testicular tissues was assessed using a TUNEL staining detection kit (Cat# C1088, Beyotime, Shanghai, China). In short, testicular tissue sections embedded in paraffin were deparaffinized, rinsed with PBS, and treated with proteinase K for permeabilization. Subsequently, the tissues were incubated in a TUNEL reaction mixture and then anti-FITC HRP was added into the tissue sections. Fluorescence microscopy (Olympus BX53, Tokyo, Japan) was utilized to capture images.

### 2.8 Immunohistochemical assays and immunofluorescence staining

The testis sections were individually deparaffinized and dehydrated using xylene and graded ethanol solutions. Antigen retrieval was carried out in a microwave oven for 20 min in a citrate buffer solution. Afterward, the slides were washed three times with TBS and then treated with bovine serum albumin (Cat# ST023, Beyotime, Shanghai, China) for 1 h at room temperature. Afterward, the testicular samples were left to incubate overnight at 4 °C in a chamber with primary antibodies targeting CBS (Cat# 14787-1-AP, Proteintech, United States) and Caspase-3 (Cat# 9661, Cell Signaling Technology, United States), then treated with secondary antibodies for 1 h at room temperature. Substrate was then applied to the sections for half an hour, followed by DAB staining. Positive immunostaining in immunohistochemistry (IHC) was primarily identified as a brownish-yellow color in the cell nuclei using a computer image analysis system. Microscopy was utilized to capture images (Olympus BX53, Tokyo, Japan).

Immunofluorescence (IF) staining was carried out as previously described (Ma et al., 2019). Briefly, the slides are fixed with 4% PFA in 4 °C refrigerator for half an hour. Afterward, the sections are incubated with the primary antibodies, N-cadherin, β-catenin, connexin-43, claudin 11, occludin and ZO-1. Secondary antibodies were used in 1:200 dilution. Utilizing fluorescence microscopy (Olympus BX53, Tokyo, Japan) to capture pictures of tissue sections with nuclei stained with DAPI (Cat# C0065, Solarbio, China).

### 2.9 Blood-testis barrier assay in testicular tissue

Based on previous studies, the EZ-Link TM Sulfo-NHS-LC-Biotin tracer (Cat# 21335, Thermo Fisher Scientific, United States) is mixed in PBS with 1 mM calcium chloride to create a working solution concentration of 10 mg/mL (Zhao et al., 2023). A 50 μL aliquot of this solution is administered via intratesticular injection into freshly dissected mouse testes. The testes are then incubated in a 37 °C water bath for half an hour, followed by fixation in 4% PFA for 24 h. In order to avoid unwanted staining, the sections are first treated with 10% goat serum for half an hour prior to being exposed to Streptavidin-Alexa Fluor 488 (Cat# S11223, Thermo Fisher Scientific, United States). DAPI was used for nuclear counterstaining. Finally, images of the testicular sections are captured using fluorescence microscopy (Olympus BX53, Tokyo, Japan).

### 2.10 Western blotting

Total protein was extracted from testicular tissues using the BCA Protein Assay kit (Cat# P0012, Beyotime, Shanghai, China). The concentration of protein was determined through this method. Once isolated, the protein was separated with sodium dodecyl sulfate polyacrylamide gel (Cat# P0012A, Beyotime, Shanghai, China), transferred to PVDF membranes, and blocked with 5% skimmed milk. Subsequently, the PVDF membranes were exposed to primary antibodies and HRP labeled secondary antibodies from Proteintech (Wuhan, China). Visualization of the protein was achieved using a chemiluminescence kit (Cat# 34577, Thermo Fisher Scientific, United States), and the intensity was calculated using Gel-Pro-Analyzer. Refer to Table 1 for the specific primary antibodies used in this study.

**TABLE 1:** Antibodies used in the study.

Antibodies	Manufacturer	Catalogue no.	Dilution
Anti-β-actin	Proteintech	66009-1-Ig	1:1000
Anti-CBS	Proteintech	14787-1-AP	1:1000
Anti-Bcl-2	Cell Signaling Tech	15,071	1:1000
Anti-Bax	Cell Signaling Tech	2,774	1:1000
Anti-Cytochrome C	Proteintech	10993-1-AP	1:1000
Anti-Cleaved Caspase-3	Abcam	ab214430	1:500
Anti-Caspase-3	Cell Signaling Tech	9,661	1:1000
Anti-Nrf2	Cell Signaling Tech	20,733	1:1000
Anti-HO-1	Cell Signaling Tech	70,081	1:1000
Anti-N-cadherin	Proteintech	22018-1-AP	1:1000
Anti-β-catenin	Bioss	bs-23663R	1:1000
Anti-ZO-1	Proteintech	21773-1-AP	1:1000
Anti-Claudin 11	Bioss	bs-2183R	1:1000
Anti-Occludin	Bioss	bs-10011R	1:1000
Anti-Connexin 43	Proteintech	26980-1-AP	1:1000
Anti-PI3K	Proteintech	20584-1-AP	1:1000
Anti-Akt	Cell Signaling Tech	9,272	1:1000
Anti-p-Akt	Cell Signaling Tech	4,060	1:1000
Anti-mTOR	Proteintech	66888-1-Ig	1:1000
Anti-p-mTOR	Proteintech	67778-1-Ig	1:1000

### 2.11 Statistical analysis

The mean ± standard deviation (SD) was utilized for the presentation of all data. Comparisons of group variances were conducted through one-way analysis of variance (ANOVA) followed by a Tukey’s *post hoc* analysis. Analysis was performed using GraphPad Prism 9.0 software. A *p*-value of less than 0.05 was deemed to be statistically significant.

## 3 Results

### 3.1 L-cysteine attenuates busulfan-induced testicular injury in mice

**FIGURE 1:**
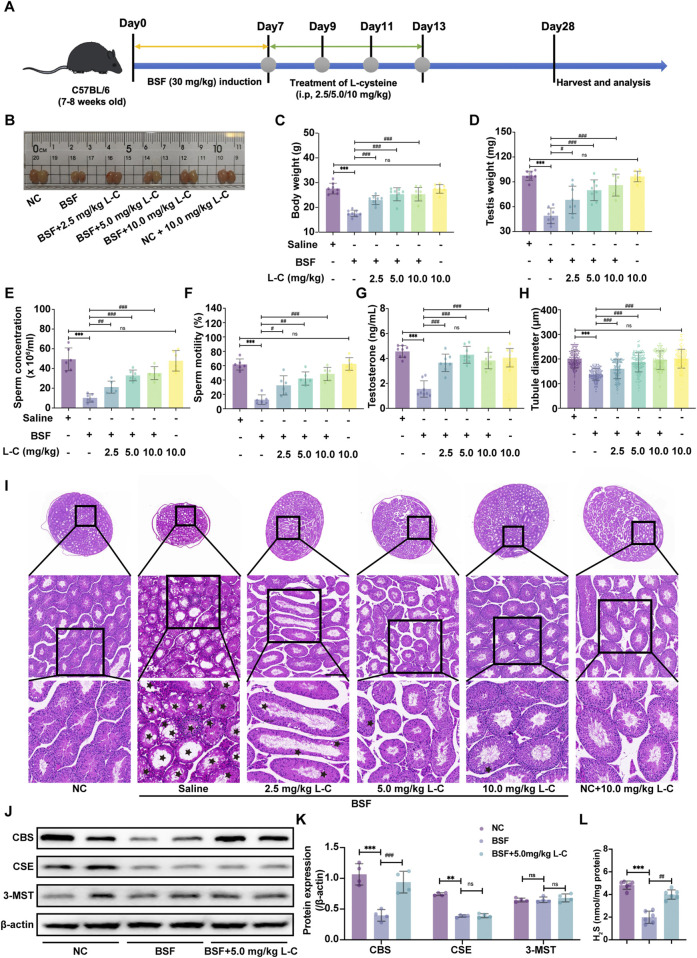
L-cysteine treatment improves the histopathologic changes in mice with busulfan-induced testicular injury. **(A)** Schematic diagram of animal experiment design. **(B)** The gross morphological changes of the testis were observed visually. **(C)** The body weight. **(D)** The testicular weight. **(E)** Sperm concentration in the caudal epididymis. **(F)** Changes in sperm motility. **(G)** Serum testosterone level. **(H)** The diameter of the seminiferous tubules. **(I)** The representative H&E staining histopathology of the testis; Scale bar: 200 μm, 100 μm and 50 μm; Black star: increased void space in the seminiferous tubules, loose cell alignment. **(J,K)** The protein levels of CBS, CSE and 3-MST was assessed by Western blotting analysis; β-actin served as the internal control. **(L)** The H2S content of testis in mice. The data were presented as the mean ± standard deviation. * and # indicate statistical significance: *p < 0.05, **p < 0.01, ***p < 0.001 compared to the NC mice group, #*p* < 0.05, ##*p* < 0.01, ###*p* < 0.001 compared to the BSF mice group.

To explore the possible benefits of H_2_S in male reproductive dysfunction, we used L-cysteine as a natural supplier of H_2_S in a mouse model with testicular toxicity induced by busulfan. Figure 1A depicted the experimental procedure visually. Results showed that giving different amounts of L-cysteine (2.5, 5, and 10 mg/kg) significantly increased the final body weight, testis weight, sperm concentration, sperm motility and testosterone levels compared to the mice group treated with busulfan (Figures 1B–G).

In order to show the positive effects of L-Cysteine in improving busulfan-induced testicular injury, the testis was analyzed using H&E histological analysis. In the BSF mice group, disrupted seminiferous tubules and disorganized germ cell layers with sloughing, detachment, and vacuolization were observed. Likewise, the sizes of circular seminiferous ducts in the BSF mice group were noticeably enlarged in comparison to the NC group. Conversely, giving L-cysteine at varying amounts decreased the testicular impairments from busulfan therapy, resulting in well-organized seminiferous tubules with orderly germinal epithelium and typical stages of epithelial cell development. The diameters of circular seminiferous tubules in the L-Cysteine-treated groups exhibited a significant increase compared to the BSF mice group (Figures 1H,I). Additionally, there were no significantly differences in reproductive organ index and sperm concentration between the NC mice group and the NC + L-C10 mice group. These observations enabled us to believe that the treatment of L-Cysteine can rescue reproductive toxicity following busulfan treatment.

To investigate the specific H_2_S-metabolizing enzymes involved in the protective effects of L-cysteine against busulfan-induced testicular injury, Western blot analysis was utilized to assess enzyme expression levels in mouse testes. Results showed that the BSF group had lower CBS and CSE expression compared to the NC group, while 3-MST expression remained unchanged. Following treatment with 5 mg/kg of L-cysteine, CBS expression increased, while CSE and 3-MST expression remained consistent. These results suggest that L-cysteine may enhance CBS expression in busulfan-induced testicular injury (Figures 1J,K). Subsequent evaluation of H_2_S levels in the testes revealed a decrease with busulfan treatment compared to controls, which was attenuated by L-cysteine (Figure 1L). Thus, it is suggested that L-cysteine may alleviate testicular injury by activating the CBS/H_2_S axis.

### 3.2 L-cysteine attenuates testicular injury via activating CBS/H_2_S axis

**FIGURE 2:**
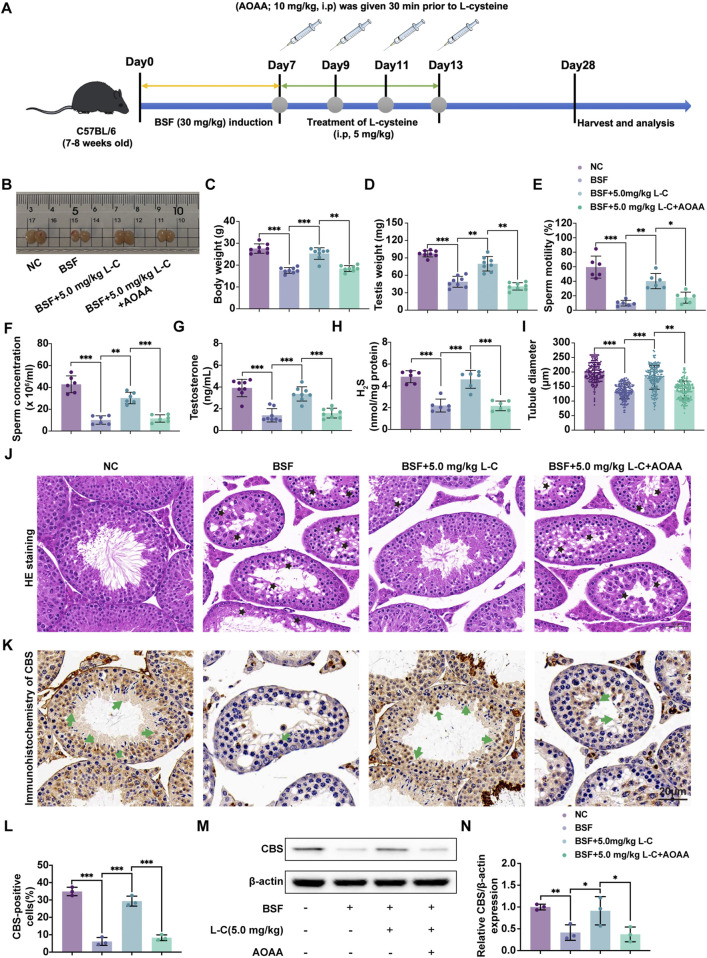
L-cysteine attenuates testicular injury via activating CBS/H2S axis. **(A)** Schematic diagram of animal experiment design following administration of L-cysteine with/without AOAA. **(B)** The gross morphological changes of the testis were observed visually. **(C)** The body weight. **(D)** The testicular weight. **(E)** Sperm concentration in the caudal epididymis. **(F)** Changes in sperm motility. **(G)** Serum testosterone level. **(H)** The H2S content in testis following administration of L-cysteine with/without AOAA. **(I)** The diameter of the seminiferous tubules. **(J)** The representative H&E staining histopathology of the testis; Scale bar: 20 μm; Black star: increased void space in the seminiferous tubules, loose cell alignment. **(K,L)** IHC were utilized to research the expression of CBS protein in seminiferous tubules and statistic results of CBS-positive cells in the four groups; Scale bar: 20 μm; Green arrows point to the positive staining. **(M, N)** The protein level of CBS was assessed by Western blot analysis; β-actin served as the internal control. The data were presented as the mean ± standard deviation. Statistical significance was determined by p-values: * for *p* < 0.05, ** for *p* < 0.01, and *** for *p* < 0.001.

To further investigate whether L-cysteine’s protective effect is mediated by the CBS/H_2_S axis, the CBS-specific inhibitor AOAA was employed to inhibit CBS activity. The experimental setup is visualized in the schematic diagram (Figure 2A). In the study, L-cysteine reversed reductions in body weight, testicular weight, sperm count, sperm motility and testosterone levels seen in BSF mice. AOAA treatment prevented the increase in these indicators (Figures 2B–G). The testicular production of H_2_S was noticeably increased in mice treated with BSF + L-Cysteine compared to those treated with just BSF, with this decrease being greatly hindered by AOAA (Figure 2H). Histological analysis using H&E staining of testicular tissue showed that L-cysteine preserved tissue integrity and tubule size, though these effects were reduced in the presence of AOAA (Figures 2I,J). Immunohistochemistry and Western blot analysis were used to assess the levels of CBS and related proteins, revealing that L-Cysteine reversed the decrease in expression caused by busulfan. This protective effect was significantly reduced after AOAA treatment (Figures 2K–N), indicating that L-Cysteine guards against busulfan-induced damage to sperm production and testicular function by activating the CBS/H_2_S pathway.

### 3.3 L-cysteine ameliorates apoptosis in mice with busulfan-induced testis toxicity

**FIGURE 3:**
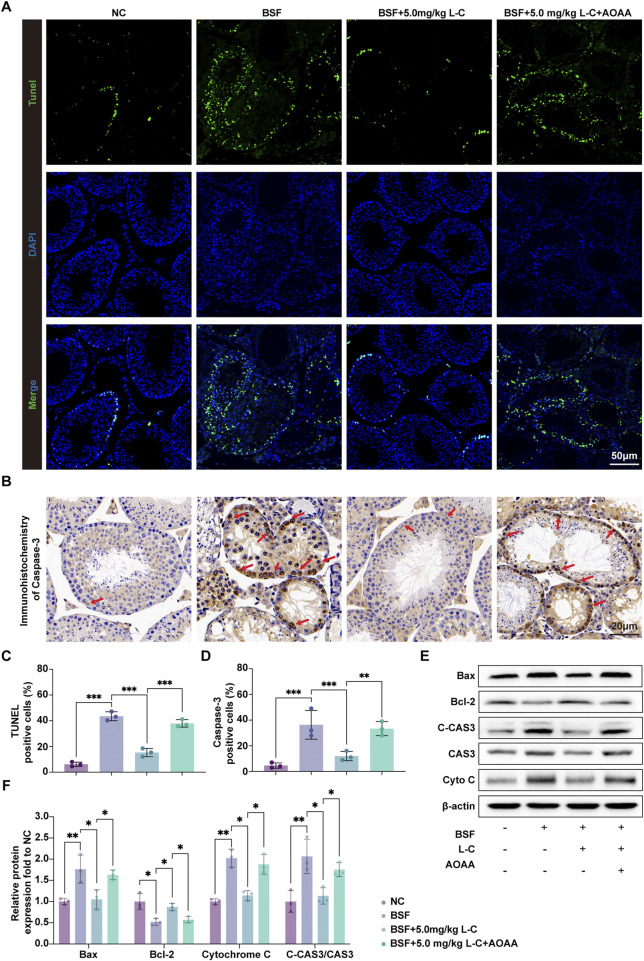
L-cysteine inhibited cell apoptosis in busulfan-induced testicular injury in mice. **(A,C)** Apoptotic cells in mouse testis tissue were stained with TUNEL staining kit and quantitative data of positive cells; Scale bar: 50 μm. **(B,D)** IHC were utilized to research the expression of Caspase-3 protein in seminiferous tubules and statistic results of Caspase-3-positive cells; Scale bar: 20 μm; Red arrows point to the positive staining. **(E,F)** The protein level of Bax, Bcl-2, Cleaved-Caspase 3, Caspase 3 and Cytochrome C was assessed by Western blot analysis; β-actin served as the internal control. The data were presented as the mean ± standard deviation. Statistical significance was determined by p-values: * for *p* < 0.05, ** for *p* < 0.01, and *** for *p* < 0.001.

To investigate the protective effects of L-cysteine on busulfan-induced testicular damage, TUNEL staining and Western blot analysis were used to assess apoptosis in different groups of mice. TUNEL staining showed a significant increase in the number of apoptotic cells in the testes of mice treated with busulfan, which was reversed by exposure to L-cysteine. Inhibition of the CBS/H_2_S axis counteracted the protective effect of L-cysteine, as evidenced by an increase in the number of apoptotic cells (Figures 3A,C). IHC results demonstrated elevated expression of Caspase-3 in the busulfan-treated group, while mice treated with L-cysteine showed a reduction in Caspase-3 levels (Figures 3B,D). Additionally, Western blot analysis revealed that L-cysteine increased the expression of anti-apoptotic protein Bcl-2, while decreasing levels of pro-apoptotic proteins Bax, Cytochrome-C and Cleaved-Caspase-3/Caspase-3 ratios in busulfan-induced testicular injury (Figures 3E,F). These findings suggest that L-cysteine effectively inhibits apoptosis in the testes following busulfan exposure.

### 3.4 L-cysteine mitigates oxidative stress in mice with busulfan-induced testis toxicity

**FIGURE 4:**
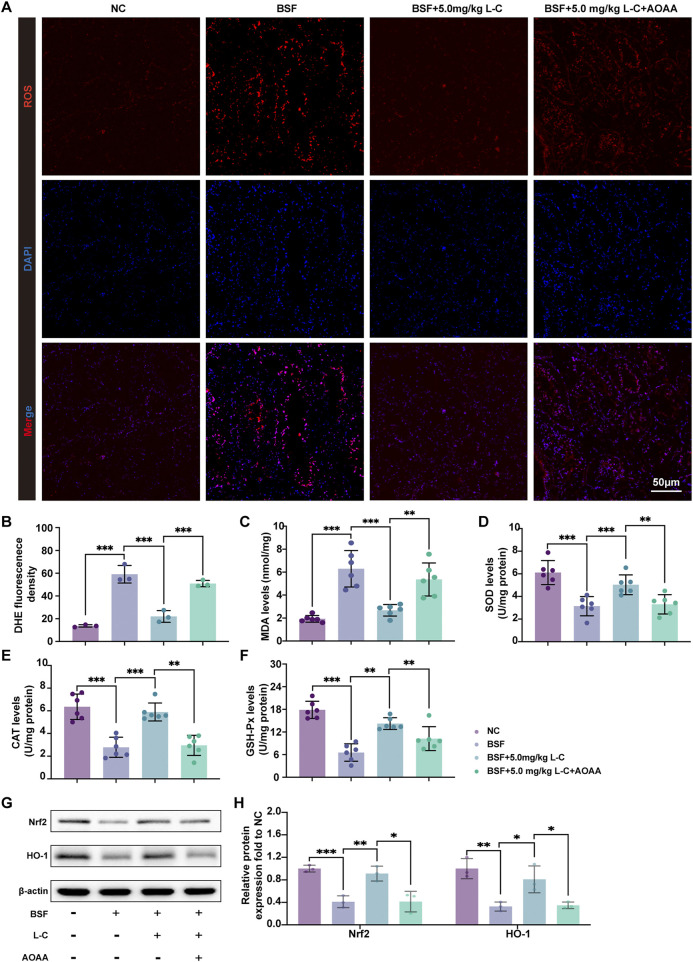
L-cysteine attenuated oxidative stress induced by busulfan in mice. **(A,B)** Images of DHE staining in testis samples; Scale bar: 50 μm. **(C–F)** Levels of MDA, SOD, CAT and GSH-Px in testis samples. **(G,H)** The protein level of Nrf2 and HO-1 was assessed by Western blotting analysis; β-actin served as the internal control. The data were presented as the mean ± standard deviation. Statistical significance was determined by p-values: * for *p* < 0.05, ** for *p* < 0.01, and *** for *p* < 0.001.

To examine the impact of L-cysteine on busulfan-caused ROS production, DHE staining of mouse testis tissue was carried out. As shown in Figures 4A,B, a marked reduction in DHE relative fluorescence intensity was observed in the BSF+L-C mice group compared with the BSF mice group in testis, and this attenuation was markedly inhibited by AOAA. SOD, CAT, and GSH-Px have the ability to neutralize deleterious free radicals and mitigate the oxidative harm inflicted on the body, while MDA serves as a marker for the degree of oxidative injury sustained by testicular tissue (Tuncer et al., 2023). The results showed that the BSF mice group exhibited decreased levels of SOD, GSH-Px, and CAT, along with an increase in MDA, in comparison to the NC mice group. Moreover, treatment with L-cysteine resulted in elevated levels of SOD, GSH-Px, and CAT, while reducing the MDA level, thereby indicating the inhibition of oxidative stress. However, these antioxidant effects were blunted by AOAA (Figures 4C–F). Besides, the study aimed to understand the molecular mechanisms underlying the antioxidant properties of L-cysteine in busulfan-induced testicular injury by examining key signaling molecules involved in ROS-mediated pathways. The results showed a significant upregulation in the protein expression levels of nuclear factor erythroid 2-related factor 2 (Nrf2) and heme oxygenase 1 (HO-1) in the BSF+L-C mice group compared to the BSF mice group. However, administration of AOAA nullified these effects (Figures 4G,H). These findings indicate that L-cysteine alleviates busulfan-induced oxidative stress in mouse testis tissue by regulating the Nrf2/HO-1 signaling pathway.

### 3.5 L-cysteine restore blood-testis barrier disruption in mice with busulfan-induced testis toxicity

**FIGURE 5:**
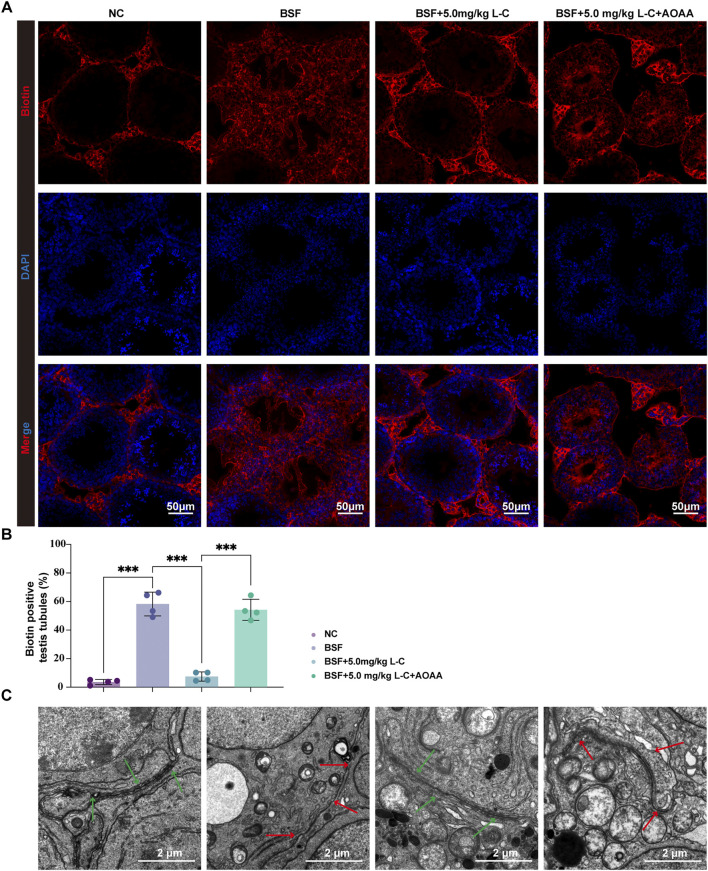
Effect of L-cysteine on busulfan induced the disruption of BTB. **(A)** Mice’s testis in experimental groups was injected with biotin, then staining is performed with streptavidin-Alexa Fluor 594 (red) and DAPI (blue) on frozen tissue sections; Scale bar = 50 μm. **(B)** Biotin positive seminiferous tubules percentage in each experimental group. **(C)** Images showing the ultrastructure of BTB as observed under transmission electron microscopy; Green arrow represented BTB structure while red arrow showed local disassembly of BTB; Scale bar: 2 μm. The data were presented as the mean ± standard deviation. Statistical significance was determined by p-values: * for *p* < 0.05, ** for *p* < 0.01, and *** for *p* < 0.001.

BTB is essential for protecting immature germ cells from the immune system and facilitating spermatogenesis (Pang et al., 2022). Busulfan-induced testicular damage often involves compromising the integrity of the BTB (Li et al., 2023; Chen et al., 2021). In this study, the protective effects of L-cysteine against BTB disruption caused by busulfan exposure were investigated. The results showed that L-cysteine effectively inhibited the diffusion of a biotin tracer in the adluminal compartment, indicating its protective role (Figures 5A,B). Afterwards, TEM was utilized to analyze the ultrastructural characteristics of BTB. As pictured in Figure 5C, continuous, bounded, tight barriers could be seen at basal Sertoli-Sertoli cell junctions in the NC and BSF+L-C mice group. Conversely, the cell junctions adjacent to Sertoli cells appeared discontinuous and disrupted in the seminiferous tubules from the BSF and BSF + L-C + AOAA mice group. These data showed that L-cysteine repaired the injured integrity of the BTB that was impaired severely by busulfan, while CBS inhibition reversed these effects.

### 3.6 Effect of L-cysteine on busulfan induced BTB components changes

**FIGURE 6:**
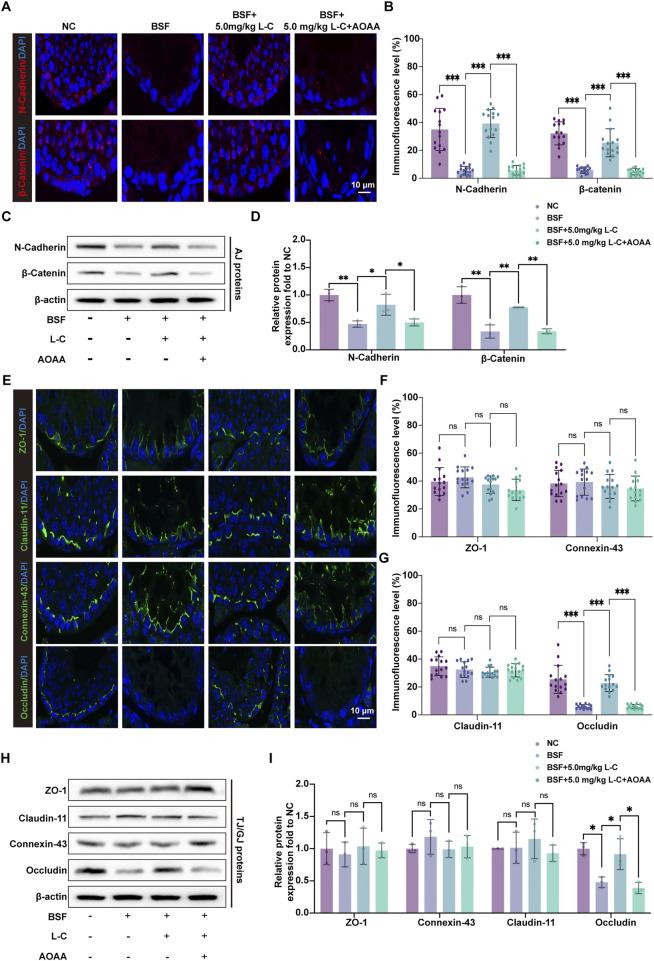
Effect of L-cysteine on busulfan induced BTB components changes. **(A)** AJ proteins were labeled using immunofluorescence with antibodies against N-Cadherin (red), β-catenin (red), and DAPI (blue) in the experimental groups; Scale bar: 10 μm. **(B)** Quantification graph of N-Cadherin and β-catenin. **(C,D)** Protein levels of N-Cadherin and β-catenin were assessed via Western blot analysis, with β-actin serving as the internal control. **(E)** Immunofluorescence staining of GJ/TJ proteins, including anti-ZO-1 (green), anti-claudin-11 (green), anti-connexin-43 (green), anti-occludin (green), and anti-DAPI (blue), was conducted in the experimental groups; Scale bar: 10 μm. **(F,G)** Quantification graph of ZO-1, claudin-11, connexin-43 and occludin immunofluorescence. **(H,I)** Western blot analysis was utilized to analyze the protein levels of ZO-1, claudin-11, connexin-43, and occludin, with β-actin as the internal reference. The data were presented as the mean ± standard deviation. Statistical significance was determined by p-values: * for *p* < 0.05, ** for *p* < 0.01, and *** for *p* < 0.001.

N-cadherin and β-catenin proteins at adherens junctions (AJs), connexin-43, claudin 11, occludin, and ZO-1 proteins at tight junctions (TJs), and gap junctions (GJs) were analyzed using IF-labeling and Western blot analysis in mouse testicular tissue. The study revealed a significant rise in IF-tagged N-cadherin and β-catenin in the BSF+L-C mice group when compared to the BSF mice group (Figures 6A,B). This observation was further supported by Western blot analysis (Figures 6C,D). Moreover, abnormal localization patterns of connexin-43, claudin 11, and ZO-1 were identified in testicular sections of the BSF mice group, accompanied by reduced occludin signals at the basal compartment of seminiferous tubules. In contrast, immunofluorescence-labeled signals of connexin-43, claudin 11, and ZO-1 maintained an organized distribution, while occludin signals were increased in testicular sections of BSF+L-C mice (Figures 6E–G). Western blot analysis demonstrated similar levels of connexin-43, claudin 11, and ZO-1 expressions, with an increase in occludin expression observed after treatment with L-cysteine compared to the BSF mice group (Figures 6H,I). Notably, supplementation with AOAA countered the beneficial effects of L-cysteine. In conclusion, our data suggests that L-cysteine may aid in restoring the BTB in busulfan-induced testicular injury.

### 3.7 Impacts of L-cysteine on activation of PI3K/Akt/mTOR pathway

**FIGURE 7:**
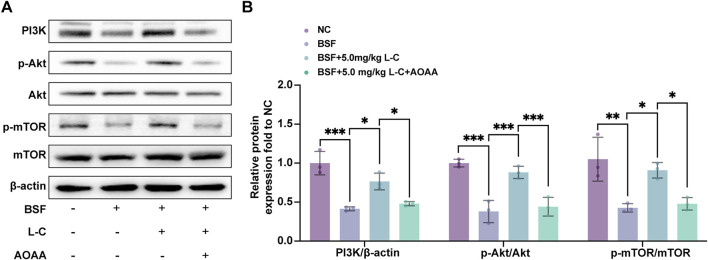
Effect of L-Cysteine on the activation of PI3K/Akt/mTOR pathway. **(A)** Western blot analysis was used to evaluate the protein levels of PI3K, p-Akt, Akt, p-mTOR, and mTOR. **(B)** Protein expression levels of PI3K, p-Akt/Akt, and p-mTOR/mTOR were quantified, with β-actin used as the internal control. The data were presented as the mean ± standard deviation. Statistical significance was determined by p-values: * for *p* < 0.05, ** for *p* < 0.01, and *** for *p* < 0.001.

The PI3K/Akt/mTOR pathway is essential for maintaining and differentiating spermatogonia, as well as regulating redox balance and metabolic activity in testicular cells to support spermatogenesis (Dhulqarnain et al., 2021; Yan et al., 2024). Previous studies have shown that H_2_S can activate this pathway to prevent cellular damage (Feng et al., 2022; Li et al., 2021). This research focused on investigating how L-cysteine affects the activation of the PI3K/Akt/mTOR pathway in testicular injury induced by busulfan. L-cysteine was found to prevent the inhibition of PI3K, as well as the phosphorylation of Akt and mTOR in testis tissue by busulfan. However, blocking CBS function using AOAA reversed the reduction of p-PI3K/PI3K ratio, p-Akt/Akt ratio, and p-mTOR/mTOR ratio induced by L-cysteine (Figures 7A,B). Overall, the data suggests that the PI3K/Akt/mTOR pathway is crucial for the protective effects of L-cysteine in preventing testicular damage caused by busulfan.

## 4 Discussion

Infertility is a prevalent health concern with global implications, affecting individuals worldwide. Male infertility constitutes fifty percent of cases, primarily attributed to sperm parameters. Hence, the main goal of this study is to explore the pharmacological actions of L-cysteine as a novel treatment for busulfan-induced testicular dysfunction. This study illustrates that supplementation of L-cysteine effectively maintains testicular function in mice treated with busulfan, as indicated by decreased testicular pathology, elevated serum testosterone levels, enhanced spermatogenesis, decreased cellular apoptosis and oxidative stress, and ameliorated disruption of BTB. Furthermore, our results indicate that L-cysteine initiates the activation of the PI3K/Akt/mTOR pathway, leading to the worsening of busulfan-induced testicular impairment. It is crucial to mention that the defense mechanisms of L-cysteine against testicular dysfunction are closely connected to the activation of the CBS/H_2_S axis.

Compelling evidence indicates that the use of busulfan leads to long-lasting damage in the testis, resulting in reduced germ cell populations and eventual shrinkage of seminiferous tubules, which hinders the production of sperm (Chen et al., 2021; Wei et al., 2020; Nakata et al., 2020). Research indicates that male pediatric cancer patients who undergo treatment regimens including busulfan often experience reduced testicular volume, significantly elevated FSH levels, and a high incidence of oligospermia or azoospermia in adulthood (Cattoni et al., 2023). Busulfan specifically targets and eradicates spermatogonial stem cells and spermatogonia that are actively proliferating (Yuan et al., 2022). During childhood, the testes are in a critical phase for the proliferation of these cells and the establishment of a cellular reserve, rendering them particularly susceptible to the effects of Busulfan (Wilhelmsson et al., 2014). The spontaneous and complete restoration of spermatogenic function is notably challenging and is typically regarded as a permanent or long-term impairment (Wang et al., 2022). Longitudinal studies have demonstrated that while certain endocrine markers may exhibit minor fluctuations over time, the majority of patients persistently display signs of spermatogenic dysfunction, such as consistently elevated FSH levels and oligospermia or azoospermia. This is particularly evident in patients who have undergone standard pre-treatment protocols for bone marrow transplantation, where such damage is generally irreversible (Gaziev et al., 2015). Comparative studies of various chemotherapy regimens have identified the gonadotoxicity associated with busulfan as particularly significant (Levi et al., 2018). In line with our findings, busulfan administration induced male reproductive harm and led to infertility, associated with decreased testicular organ coefficients, reduced serum testosterone levels, and significant testicular histopathological alterations.

H_2_S is a recently discovered endogenous signaling gasotransmitter that plays a role in various physiological and pathological processes within the male reproductive system (Wang et al., 2018; Li et al., 2022; Mao et al., 2023). L-cysteine, possessing a sulfhydryl group at the terminus of its side chain, exhibits reactivity (La Fuente et al., 2019; Farace et al., 2019). Through the generation of H_2_S, L-cysteine can indirectly modulate cellular responses (Aydinoglu et al., 2017; Yin et al., 2016). Administration of L-cysteine at varying dosages (2.5, 5.0, or 10.0 mg/kg) demonstrated efficacy in ameliorating busulfan-induced testicular dysfunction, as evidenced by reduced morphological damage in testicular histology, increased body weight, testicular weight, and testosterone levels. Enzymes like CBS, CSE, and 3-MST catalyze the generation of H_2_S from L-cysteine (Hsu et al., 2018; Bronowicka-Adamska et al., 2017). During the current study, it was discovered that administering busulfan resulted in a reduction in the levels of CBS and CSE in the testicular tissues of mice, with no change observed in the expression of 3-MST. Interestingly, L-Cysteine specifically maintained the expression of CBS following busulfan treatment, resulting in an increase in H_2_S production. AOAA, acting as a CBS inhibitor, successfully reversed the negative effects of L-Cysteine on testicular damage and the increase in CBS expression after busulfan treatment. This indicates that the beneficial impact of L-Cysteine on sperm production is mainly due to the preservation of CBS expression for the production of H_2_S. According to the information provided, L-Cysteine could have a positive impact on protecting reproduction from busulfan-induced testicular damage in mice. Nevertheless, more research is needed to understand how it works.

Apoptosis, a common type of cell death that is programmed, is triggered by the rise in mitochondrial permeability caused by reactive oxygen species. Cytochrome C is able to move to the cytoplasm, leading to the initiation of the caspase apoptotic pathway and eventually resulting in the activation of apoptotic effector caspase-3 (Wang et al., 2014; Aly and Khafagy, 2014). Busulfan-induced reproductive toxicity, characterized by increased DNA damage and enhanced cell apoptosis, was observed in the testis (Qian et al., 2020). In our research, the administration of L-Cysteine demonstrated a significant reduction in the apoptotic response within testicular tissue following exposure to busulfan.

The pathogenesis of busulfan-induced testicular injuries is closely associated with oxidative stress-induced damage, perturbations in the testis antioxidant defense system, and the production of ROS (Abarikwu et al., 2022b). H_2_S demonstrates notable antioxidant and radical scavenging properties within cellular compartments, including the cytoplasm and nucleus (Guidotti et al., 2023; Hu et al., 2022; Qabazard et al., 2020). These effects are achieved through the activation of robust free radical scavenging mechanisms, upregulation of antioxidant enzymes and preservation of antioxidant enzyme integrity against oxidative stress, which is crucial for regulating reactive oxidative species production (Munteanu et al., 2023; Retracted, 2023; Zhu Z. et al., 2022; Xu et al., 2022). The study’s results indicated that L-cysteine effectively reduced levels of ROS and MDA while enhancing the activities of GSH-PX, SOD, and CAT in busulfan-induced testicular injuries. This implies that L-cysteine may aid in restoring oxidative stress balance by enhancing antioxidant enzyme functionality. HO-1, an Nrf2-regulated gene, is a crucial target for antioxidants as part of the Nrf2/HO-1 pathway. Increasing evidence suggests that H_2_S offers antioxidant protection against various diseases by modulating the Nrf2/HO-1 pathway (Hamidizad et al., 2022; Waz et al., 2021; Chen et al., 2019). Our investigation discovered that L-cysteine significantly boosted Nrf2/HO-1 pathway activation in busulfan-induced testicular damage compared to the busulfan-only treated group, indicating a potential antioxidant role for L-cysteine.

BTB serves as a critical protective barrier in the male reproductive system, separating the seminiferous tubules from the capillaries to safeguard the microenvironment essential for spermatogenesis. Biotin tracing analysis indicated that the permeability of the BTB was increased in busulfan-induced testicular dysfunction and could be ameliorated with L-cysteine. TJs are a critical element of the BTB, regulating the passage of metabolites through selective permeability. The key structural proteins of TJs include claudin 11, occludin and ZO-1. Within the seminiferous epithelium, connexin43 is the predominant GJs protein, facilitating direct cytoplasmic communication between adjacent testicular cells and contributing to the maintenance of BTB homeostasis through its influence on tight junction reassembly, thereby playing a crucial role in mammalian spermatogenesis. It is reported that busulfan directly disrupts BTB integrity by disturbing the expression and localization of TJs-related proteins and GJs-related protein, leading to spermatogenic dysfunction and infertility (Liu et al., 2021; Zhao et al., 2022; Li et al., 2023). Our study suggested that L-cysteine may reverse busulfan-induced abnormal expression and localization of claudin 11, connexin-43, ZO-1 and occludin in the BTB. AJs also play a crucial role in the BTB, with various reproductive toxicity agents, such as plastic particles, bisphenol-A, and melamine, known to disrupt AJs and impact the barrier system (Chang et al., 2018; Marcelino et al., 2022; Zhu L. et al., 2022). Additionally, exposure to microcystin-leucine arginine has been shown to impair AJs by downregulating the expressions of β-catenin and N-Cadherin, leading to BTB damage and compromised male reproductive function (Liu et al., 2022). Importantly, the present study revealed that busulfan exposure reduced the expression of N-Cadherin and β-catenin in mouse testes, whereas these protein expressions were largely restored upon L-cysteine treatment. The above findings suggest collectively suggest that L-cysteine may support spermatogenesis by maintaining the structural integrity of the BTB, potentially enhancing spermatogenesis by safeguarding spermatogonia and Sertoli cells.

The crucial role of the PI3K/Akt/mTOR pathway in intracellular signaling cannot be overstated, as it regulates cell function and apoptosis. Studies have shown that this pathway is essential for the proper functioning of the male reproductive system, influencing processes such as testicular cell survival, cell death, cell morphology, redox balance, and metabolic regulation to ensure functionality (Luo et al., 2020; Wang et al., 2020; Deng et al., 2021; Fu et al., 2020). Environmental endocrine disruptors, including bisphenol A and its analogs, have been demonstrated to impair spermatogenesis through the inhibition of the PI3K/Akt/mTOR signaling pathway, while concurrently inducing autophagy and apoptosis in testicular cells (Wu et al., 2021; Wang et al., 2025). Analogously, exposure to phthalates, particularly di (2-ethylhexyl) phthalate (DEHP), has been shown to activate the PI3K/Akt/mTOR pathway in the testes of both F1 and F2 generation mice, thereby causing transgenerational reproductive harm (Fu et al., 2020). Moreover, heavy metals such as cadmium, alongside emerging concerns regarding nanoparticles like titanium dioxide nanoparticles, have been implicated in the induction of oxidative stress (Wang et al., 2020; Hong and Zhou, 2020). This oxidative stress results in the inhibition of the PI3K/Akt/mTOR pathway, leading to apoptosis of spermatogenic cells, excessive autophagy, and disruption of the BTB. Notably, certain natural compounds have exhibited potential in mitigating these toxic effects. For instance, ginsenoside Rg3 has been found to ameliorate male reproductive dysfunction induced by dibutyl phthalate and monobutyl phthalate by reactivating the PI3K/Akt/mTOR pathway (Li et al., 2025). Similarly, melatonin confers protection against DEHP-induced prepubertal testicular injury via modulation of the PI3K/Akt/mTOR pathway (Chen et al., 2024). Additionally, disruptions in the PI3K/Akt/mTOR pathway have been linked to the maintenance of the BTB (Chen et al., 2022; Zhang et al., 2023). However, the effects of L-cysteine-induced activation of the PI3K/Akt/mTOR pathway on busulfan-induced testicular damage are not yet fully understood. Our findings indicate that treatment with L-cysteine resulted in increased levels of PI3K, a higher p-Akt/Akt ratio, and an elevated p-mTOR/mTOR ratio in response to busulfan-induced injury. Interestingly, AOAA treatment effectively mitigated these heightened levels of signaling factors.

Several limitations and critical questions warrant further investigation in future research. The primary limitation arises from the reliance on a mouse model. Although mice are widely utilized as a standard in reproductive toxicology studies, inherent interspecies differences in physiology and developmental processes, such as the timing of spermatogenesis and embryonic development, constrain the direct extrapolation of these findings to human clinical outcomes. Additionally, while our data indicate that L-cysteine mitigates busulfan-induced damage through the CBS/H_2_S axis and is associated with the activation of the PI3K/Akt/mTOR pathway, this study is correlational and does not definitively establish a direct causal relationship between these mechanisms. The protective effects of L-cysteine are likely pleiotropic, potentially involving other, as yet unexplored, signaling pathways.

In future research, it is imperative to address the existing limitations through several critical approaches. Firstly, a more detailed, cell-specific mechanistic analysis is necessary. Utilizing isolated testicular cell cultures, such as Sertoli and Leydig cells, could elucidate how L-cysteine directly influences the PI3K/Akt/mTOR pathway and its subsequent effects on cellular function and viability. Additionally, future studies should investigate the long-term effects, determining whether the protective benefits of L-cysteine are sustained over time and assessing its impact on other organs vulnerable to busulfan toxicity, thereby ensuring a comprehensive therapeutic advantage. These steps are essential for translating our preclinical findings into a feasible clinical strategy for fertility preservation.

## 5 Conclusion

Our study demonstrates that L-cysteine can alleviate busulfan-induced testicular injury in mice by reversing changes in body weight, testis weight, sperm concentration, and testicular histology, all of which are linked to the activation of the CBS/H_2_S axis. The protective effects of L-cysteine on busulfan-induced apoptosis, oxidative damage, and BTB injury are supported by the restoration of the PI3K/Akt/mTOR pathway. This investigation represents the initial investigation into the protective properties of L-cysteine against busulfan-induced testicular damage and the underlying mechanisms, providing a theoretical and empirical basis for addressing male reproductive health concerns. Future research is necessary to corroborate the mechanisms elucidated in this study.

## Data Availability

The datasets presented in this article are not readily available because Processed data are available from the corresponding author upon reasonable request. Requests to access the datasets should be directed to sdsfyliusong@163.com.
